# The Effect of Laparoscopic Sleeve Gastrectomy on the Course of Non-Alcoholic Fatty Liver Disease in Morbidly Obese Patients during One Year of Follow Up

**DOI:** 10.3390/jcm12124122

**Published:** 2023-06-18

**Authors:** Paulina Głuszyńska, Aleksander Łukaszewicz, Inna Diemieszczyk, Jan Chilmończyk, Joanna Reszeć, Anna Citko, Łukasz Szczerbiński, Adam Krętowski, Hady Razak Hady

**Affiliations:** 1 Department of General and Endocrine Surgery, Medical University of Bialystok, 15-276 Bialystok, Poland; alexander.luk6@gmail.com (A.Ł.); janchilmonczyk@gmail.com (J.C.); hadyrazakh@wp.pl (H.R.H.); 2 Department of Surgery, Independent Public Health Care Center in Lapy, 18-100 Lapy, Poland; demeschik.inna@gmail.com; 3 Department of Medical Pathomorphology, Medical University of Bialystok, 15-269 Bialystok, Poland; joasia@umb.edu.pl; 4 Clinical Research Centre, Medical University of Bialystok, 15-276 Bialystok, Poland; anna.citko@umb.edu.pl (A.C.); lukasz.szczerbinski@umb.edu.pl (Ł.S.); adam.kretowski@umb.edu.pl (A.K.); 5 Department of Endocrinology, Diabetology and Internal Diseases, Medical University of Bialystok, 15-276 Bialystok, Poland

**Keywords:** bariatric/metabolic surgery, laparoscopic sleeve gastrectomy, non-alcoholic fatty liver disease, morbid obesity

## Abstract

Background: Morbid obesity co-exists with non-alcoholic fatty liver disease in up to 90% of cases. Laparoscopic sleeve gastrectomy leads to a reduction in body mass and thus may improve the course of non-alcoholic fatty liver disease. The aim of this study was to evaluate the effect of laparoscopic sleeve gastrectomy on the resolution of non-alcoholic fatty liver disease. Methods: The study included 55 patients with non-alcoholic fatty liver disease who underwent laparoscopic sleeve gastrectomy at a tertiary institution. The analysis consisted of preoperative liver biopsy, abdominal ultrasound, weight loss parameters, Non-Alcoholic Fatty Liver Fibrosis Score and selected laboratory parameters. Results: Before the surgery, 6 patients were diagnosed with grade 1 liver steatosis, 33 patients with grade 2 and 16 patients with grade 3. One year after the surgery, only 21 patients had features of liver steatosis at ultrasound. All weight loss parameters showed statistically significant changes during the observation; the median percentage of total weight loss was 31.0% (IQR: 27.5; 34.5) with *p* = 0.0003, the median percentage of excess weight loss was 61.8% (IQR: 52.4; 72.3) with *p* = 0.0013 and the median percentage of excess body mass index loss was 71.0% (IQR: 61.3; 86.9) with *p* = 0.0036 12 months after laparoscopic sleeve gastrectomy. The median Non-Alcoholic Fatty Liver Fibrosis Score at baseline was 0.2 (IQR: −0.8; 1.0) and decreased to −1.6 (IQR: −2.4; −0.4) (*p* < 0.0001). Moderate negative correlations between Non-Alcoholic Fatty Liver Fibrosis Score and percentage of total weight loss (r = −0.434, *p* < 0.0001), percentage of excess weight loss (r = −0.456, *p* < 0.0001) and percentage of excess body mass index loss (r = −0.512, *p* < 0.0001) were found. Conclusions: The study supports the thesis that laparoscopic sleeve gastrectomy is an effective method for treatment of non-alcoholic fatty liver disease in patients with morbid obesity.

## 1. Introduction

The pandemic of obesity has become a serious issue of public health worldwide as the size of the obese population has almost tripled over the last four decades and continues to rise [1]. This HAS resulted in a significant increase in the prevalence of non-alcoholic fatty liver disease (NAFLD). NAFLD is currently the most common chronic liver disease, with an estimated global prevalence at 25–30%, rising up to 90% in morbidly obese patients [2]. According to US guidelines, NAFLD is recognized when there is ≥ 5% steatotic hepatocytes in imaging or histology with no alcohol-, drug- or viral-induced steatosis [3]. The spectrum of NAFLD ranges from benign hepatocellular steatosis to non-alcoholic steatohepatitis (NASH), fibrosis and eventually cirrhosis and may lead to the development of hepatocellular carcinoma (HCC). It is believed that one third of patients at an early stage of NASH will progress to fibrosis within 5 to 10 years after the diagnosis. Considering indications for liver transplant, NAFLD/NASH is currently the most rapidly growing cause of HCC among patients on the waiting list in the United States, increasing from 2.1% in 2002 to 16.2% in 2016 (*p* < 0.0001) [4]. According to the US National Liver Transplantation Registry from 2018, 34.6% of liver transplant recipients had a BMI >3 0 kg/m^2^, and almost 14% had a BMI > 35 kg/m^2^ [5]. The main management option for obesity-related NAFLD is weight reduction by 7–10% with lifestyle modifications including dietary changes and physical activity. However, this goal may be difficult to achieve in obese patients and even more problematic to maintain. Studies have shown that more than 90% of obese patients cannot achieve this target during one year of observation [6,7]. Bariatric surgery is an option for obese individuals who fail to achieve suitable weight loss with lifestyle changes and pharmacological methods. Bariatric surgery can help obese individuals achieve recommended weight reduction and thus improve the course of NAFLD. The additional benefits of bariatric surgery include resolution or amelioration of hypertension, hyperlipidemia and type 2 diabetes and reduction of cardiovascular risk and mortality [8,9]. One of the most commonly performed bariatric procedures worldwide is laparoscopic sleeve gastrectomy (LSG). The IFSO Global Registry 2018 Report provided data from 51 different countries; data were reported on 87,467 sleeve gastrectomy operations (46.0%), 72,645 Roux-en-Y gastric bypass operations (38.2%), 14,516 one-anastomosis gastric bypass procedures (7.6%) and 9534 gastric banding operations (5.0%) [10]. LSG reduces stomach volume and also causes a decrease in ghrelin level, which is also called “a hormone of appetite” [11,12]. The following study aims to show changes in the course of NAFLD in morbidly obese patients undergoing laparoscopic sleeve gastrectomy in one year of observation and support the thesis that the above-mentioned bariatric procedure is an effective method for treating the liver manifestation of metabolic syndrome.

## 2. Materials and Methods

**Figure 1:**
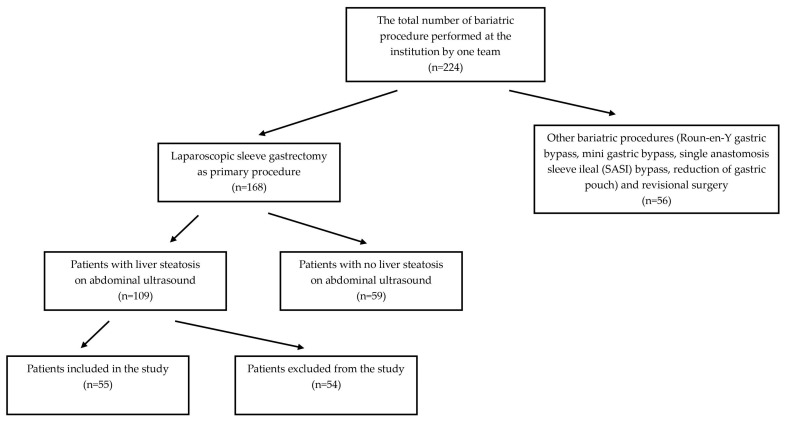
Graphical guidelines for study group selection.

This is a retrospective study of patients who underwent laparoscopic sleeve gastrectomy and were diagnosed with liver steatosis in abdominal ultrasound prior to the surgery. The procedures were performed in the University Hospital at a tertiary institution between 2019 and 2021. Patients were qualified for surgical treatment of morbid obesity according to the Polish Guidelines on Metabolic and Bariatric Surgery [13]. The inclusion criteria for the surgical procedure comprised inability to achieve sustained weight loss with conservative management and BMI ≥ 40.0 kg/m^2^ or 35–40 kg/m^2^ with the presence of at least one obesity-related co-morbidity such as type 2 diabetes mellitus or insulin resistance, hypertension, dyslipidemia, obstructive sleep apnea, non-alcoholic fatty liver disease and non-alcoholic steatohepatitis, osteoarthritis, coronary artery disease and infertility in women resulting from polycystic ovary syndrome. Patients with obesity-related endocrine diseases, clinically significant or unstable mental health concerns and addiction to alcohol or psychostimulants and women planning on pregnancy within two years after a potential surgery were excluded from the surgical procedure. Study inclusion criteria: patients who underwent LSG as a primary obesity surgery, patients with diagnosed NAFLD based on abdominal ultrasound and no additional procedures during laparoscopic sleeve gastrectomy. The approximate time between diagnosis of NAFLD and bariatric procedure was 6 months. Exclusion criteria were viral hepatitis, autoimmune hepatitis, hemochromatosis, alcoholic liver cirrhosis and complications during the surgery or observation period. Patients were also excluded from the study when there was a lack of necessary data. Figure 1 presents the explanation of the ultimate definition of the study group.

Demographic and clinical data were gathered before the surgery, as well as 6 and 12 months after the bariatric procedure. Postoperative weight loss was expressed in terms of percent total weight loss (%TWL), percent excess weight loss (%EWL) and percent excess BMI loss (%EBMIL). The following equations were used:

- Percent total weight loss: %TWL = (initial weight-current weight)/(initial weight) × 100;
- Percent excess BMI loss: %EBMIL = (initial BMI-postoperative BMI)/(initial BMI-25) × 100;
- Percent excess weight loss: %EWL = (initial weight-postoperative weight)/(initial weight-ideal weight) × 100, where ideal weight is defined by the weight corresponding to a BMI of 25 kg/m^2^.

Biochemical analysis included aspartate aminotransferase (AST), alanine aminotransferase, (ALT), gamma-glutamyl transpeptidase (GGT), lactate dehydrogenase (LDH), bilirubin, serum albumin, fasting glucose level, platelet count, total cholesterol, triglyceride, HDL cholesterol and LDL cholesterol levels.

Advanced hepatic fibrosis was assessed by the Non-Alcoholic Fatty Liver Disease Fibrosis Score (NAFLD Fibrosis Score). The calculation was performed according to the following formula:

NAFLD Fibrosis Score = −1.675 + 0.037 × age (years) + 0.094 × BMI (kg/m²) + 1.13 × hyperglycemia/diabetes (yes = 1, no = 0) + 0.99 × AST/ALT ratio − 0.013 × platelets (× 10⁹/L − 0.66 × albumin, g/dL). Values below −1.455 were considered as the absence of liver fibrosis and those above 0.676 as the presence of advanced hepatic fibrosis. Values between −1.455 and 0.676 were considered as indeterminate hepatic fibrosis [14].

Abdominal ultrasound was performed before the surgical procedure and 6 and 12 months after the surgery. Liver steatosis in abdominal ultrasound was graded as follow:

- Score 0 (absent)—normal echotexture of the liver;
- Score 1 (mild)—a slight and diffuse increase in liver echogenicity with normal visualization of the diaphragm and of the portal vein wall;
- Score 2 (moderate)—a moderate increase in liver echogenicity with slightly impaired appearance of the portal vein wall and the diaphragm;
- Score 3 (severe)—marked increase in liver echogenicity with poor or no visualization of portal vein wall, diaphragm and posterior part of the right liver lobe.

The hepatic biopsy was performed during the laparoscopic sleeve gastrectomy. Histopathological examination included the assessment of the presence or absence of steatosis, fibrosis and lobular inflammation.

### 2.1. Surgical Technique

The greater curvature of the stomach was dissected starting by 6 cm to the pylorus up to the His angle. The reduction in stomach volume was performed using a 36-Fr bougie and 60 mm linear staplers. At the end, the leak test was performed with the use of methylthioninium chloride solution and air. The gastric specimen was sent to pathology examination. Patients were discharged home a day after the surgery if no complications occurred.

### 2.2. Data Analysis

Data were analyzed using GraphPad Prism 9.0.0 software (GraphPad Software, San Diego, CA, USA). Normality of distribution was checked by the W Shapiro–Wilk test. The Wilcoxon matched-pairs signed-rank test was used for comparison between the two groups. The ANOVA Friedmann test was applied to comparisons between more than two groups and the paired Dunn’s test for post hoc analysis. Continuous values are presented as medians with interquartile ranges. The correlation between examined parameters and the strength of that relationship was measured with the nonparametric Spearman rank-order correlation coefficient. The significance level was set at *p* < 0.05.

## 3. Results

**Figure 2:**
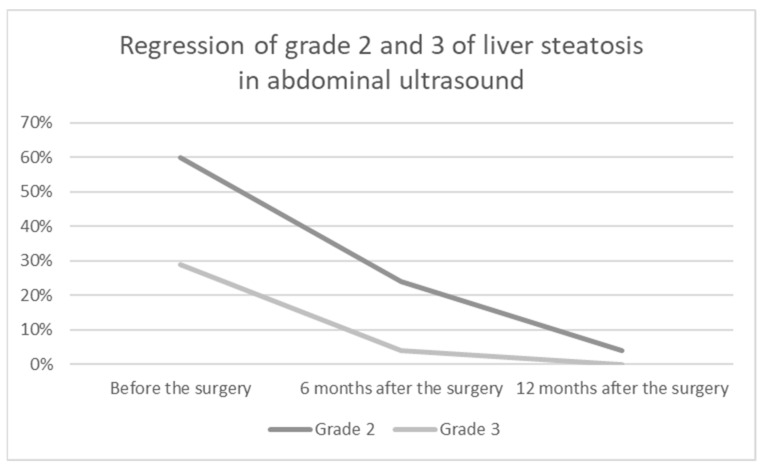
Graphical presentation of liver steatosis regression in abdominal ultrasound.

The study group included 55 patients, 32 men (58%) and 23 women (42%). The median age of patients at the time of surgery was 43.5 years (22–54 years). The median preoperative BMI was 45.6 (IQR: 42.5; 50.2) kg/m^2^. Of the patients, 62% (*n* = 34) had hypertension, 27% insulin resistance or type 2 diabetes (*n* = 15) and 41% hypercholesterolemia (*n* = 23). Preoperatively, 6 patients were diagnosed with grade 1 liver steatosis, 33 patients with grade 2 and 16 patients with grade 3. One year after the surgery, only 21 patients had features of liver steatosis in abdominal ultrasound—grade 1 was observed in 19 patients and grade 2 in 2 patients. The assessment of liver steatosis and its changes in abdominal ultrasound during one year of observation is presented in Table 1 and Figure 2. The analysis of preoperative liver specimens revealed hepatic steatosis in all patients, inflammatory features in 32 patients (58.2%) and liver fibrosis in 12 patients (21.8%).

**Figure 3:**
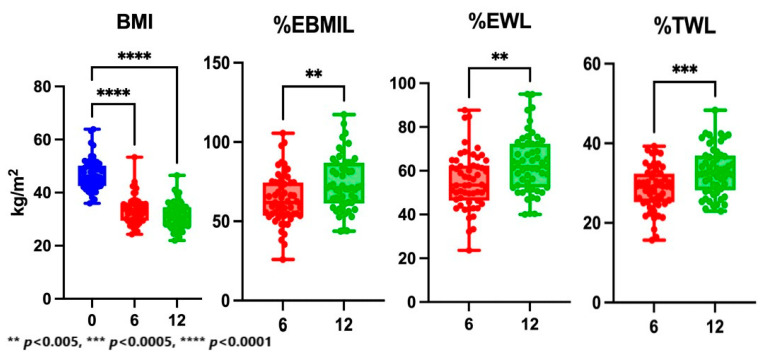
Changes in weight loss parameters during the observation. BMI, body mass index; %EBMIL, percentage of excess BMI loss; %EWL, percentage of excess weight loss; %TWL, percentage of total weight loss. Blue color refers to the preoperative examination, red—6 months after the surgery and green—12 months after the surgery.

All parameters representing postoperative weight loss showed a statistically significant increase in one year of observation. The median %EBMIL rose from 61.8% (IQR: 53.6; 74.4) 6 months after the surgery to 71.0% (IQR: 61.3; 86.9) 12 months after the bariatric procedure (*p* = 0.0036). The median %EWL increased to 61.8% (IQR: 52.4; 72.3) with *p* = 0.0013 and median %TWL to 32.5% (IQR: 28.2; 36.9) with *p* = 0.0003 one year after the bariatric procedure. The results of bariatric effect in the study group are presented in Table 2 and Figure 3.

The amelioration in liver enzymes profile was observed in one year of follow up, including AST (25.5 (IQR: 19.0; 37.0) vs. 20.0 (IQR: 17.0; 26.0)), ALT (41.10 (IQR: 21.0; 53.9) vs. 19.0 (IQR: 16.0; 24.0)), GGT (28.5 (IQR: 21.6; 56.5) vs. 18.0 (IQR: 13.7; 35.0)) and LDH (235.0 (IQR: 186.0; 271.0) vs. 176.0 (IQR: 152.0; 184.0)). Table 3 presents changes in selected laboratory parameters and NAFLD Fibrosis Score during the observation.

**Figure 4:**
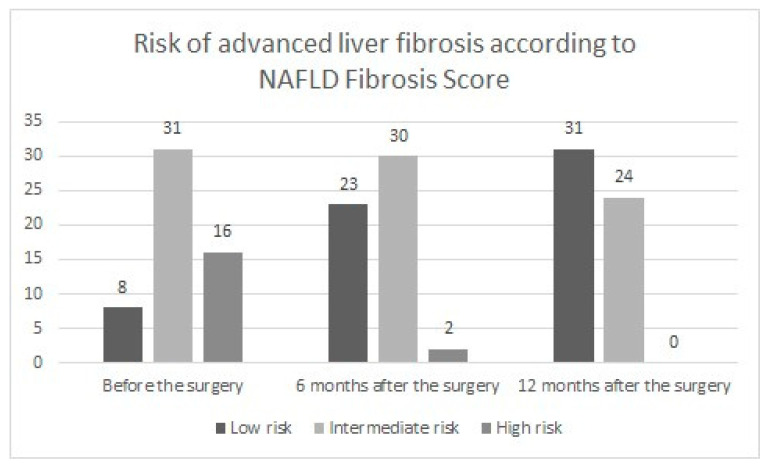
Risk of advanced hepatic fibrosis based on NAFLD Fibrosis Score.

The median NAFLD Fibrosis Score at baseline was 0.2 (IQR: −0.8; 1.0) and decreased to −1.6 (IQR: −2.4; −0.4) one year after the surgery (*p* < 0.0001). There was a negative moderate correlation between NAFLD Fibrosis Score and mean %TWL (r = −0.434, *p* < 0.0001), %EWL (r = −0.456, *p* < 0.0001) and %EBMIL (r = −0.512, *p* < 0.0001). The assessment of the risk of advanced liver fibrosis and its changes during the observation is presented in Figure 4.

## 4. Discussion

This study investigated the impact of one of the bariatric procedures, laparoscopic sleeve gastrectomy, on the course of non-alcoholic fatty liver disease during one year of observation.

Despite a number of promising treatment options for NAFLD, including antidiabetic and anti-obesity drugs, drugs modifying the lipid profile, vitamin E supplementation and novel therapeutic treatments inclusive of medication that interfere with inflammatory, fibrotic and apoptotic pathways, healthy lifestyle modification combined with a decrease in body mass remains at the core of management of NAFLD and NASH [15]. Dietary recommendations for individuals with obesity and non-alcoholic fatty liver disease include: reduction in energy intake, reduction in fructose consumption and a well-balanced diet comprising 40–50% energy from carbohydrates, ≤30% fat (saturated fatty acids >7% and <10% total energy) and about 20% protein [16]. However, very often, the above recommendations are difficult to fulfill, and obese patients fail to achieve the expected weight loss. Several studies have shown that laparoscopic sleeve gastrectomy causes significant weight loss over both short- and long-term observation periods [17,18,19]. Kraljević et al. analyzed 307 patients who underwent LSG as a primary bariatric procedure. The mean %EBMIL was 62.8 ± 23.1% after 5 years, 53.6 ± 24.6% after 10 years and 51.2 ± 20.3% after 13 years [20]. Our study also proved that laparoscopic sleeve gastrectomy contributes to considerable body mass reduction in patients with morbid obesity, reaching a median %EBMIL of 71.0% (IQR: 61.3; 86.9) after 12 months. Algooneh et al. analyzed the impact of %EWL on the resolution of NAFLD. A significant resolution of NAFLD was seen in patients achieving a mean %EWL > 50% (OR 10.1; *p* < 0.001). However, resolution of NAFLD was observed even in patients with a mean %EWL of 30% (OR 7.0, *p* = 0.024) [21]. In this study, the median percentage of excess weight loss reached 61.8% (IQR: 52.4; 72.3) one year after laparoscopic sleeve gastrectomy.

In a study conducted by Mattar et al., it was observed that weight loss induced by bariatric surgery (Roux-en-Y gastric bypass (RYGB) or LSG) causes significant improvement or resolution of NAFLD and NASH in liver histology, including steatosis, inflammation and fibrosis [22]. Fakhry et al. conducted a wide metanalysis that included 21 studies with a total number of 2374 patients who had undergone bariatric surgery (vertical-banded gastroplasty (VGB), laparoscopic adjustable gastric banding (LAGB), RYGB or LSG). They provided strong evidence that bariatric surgery not only improves biochemical and histological features of NAFLD but also terminates the progression of the disease and resolves it in up to 30% of patients [23]. In our study, the total resolution rate for liver steatosis in abdominal ultrasound was 62% (34 patients) one year after laparoscopic sleeve gastrectomy.

Bower et al. conducted a systematic review and proved that bariatric surgery is associated with improvement of the histological features of NAFLD, including steatosis (50.2 and 95%CI of 35.5–65.0), fibrosis (11.9 and 95% CI of 7.4–16.3%) and lobular inflammation (50.7 and 95% CI, 26.6–74.8%) [24]. Another metanalysis that included 32 cohort studies comprising 3093 biopsy specimens showed that bariatric surgery is an effective method for the treatment of NAFLD, resulting in biopsy-confirmed resolution of steatosis in 66% patients (95% CI, 56–75%), inflammation in 50% (95% CI, 35–64%), ballooning degeneration in 76% (95% CI, 64–86%) and fibrosis in 40% (95% CI, 29–51%). However, this metanalysis showed new features or worsening of NAFLD in 12% (95% CI, 5–20%) of patients [25]. Moretto et al. analyzed 78 morbidly obese patients who had undergone gastric bypass and had undergone liver biopsy during the surgery and after weight loss. They found that the prevalence of liver fibrosis was 44.9% (CI 95% 33.6–56.6%) at the first biopsy and 30.8% (CI 95% 20.8–42.2%) after weight loss (*p* = 0.027) [26]. However, it is also known that rapid weight loss may increase the risk of hepatic fibrosis. Weight loss of more than 1.6 kg per week results in a rapid reduction in hepatic fat and a subsequent increase in visceral free fatty acids and proinflammatory cytokines, which may worsen the course of the histological features of NAFLD [27]. An interesting observation was made by Mathurin et al. Their research showed that the improvement of steatosis and ballooning occurred mainly during the first year after bariatric surgery and persisted up to 5 years postoperatively. However, they noticed that liver fibrosis worsened at 5 years even though more than 95% of patients had a Fibrosis Score ≤ F1 [28]. The research conducted by Mottin et al. showed that 16 out of 90 patients (17.8%) who underwent bariatric surgery had the same degree of liver steatosis at the second biopsy as during the operation [29].

A study conducted by Ruiz-Tover et al. showed that liver steatosis measured by abdominal ultrasound improves after sleeve gastrectomy. A complete resolution in liver steatosis was observed in 90% of patients included in their study [30]. Complete resolution measured by ultrasonography in our study was seen in 62% of all patients. Another study conducted by Elyasinia et al. proved that both laparoscopic sleeve gastrectomy and gastric bypass significantly enhance hepatic status in ultrasonography. Preoperatively, 81.8% of patients were diagnosed with grade I or II liver steatosis. One year after the surgery, 72.7% of patients presented no NASH signs in ultrasonography [31]. According to our study, 19 patients (34.5%) had grade 1 liver steatosis in abdominal ultrasonography after one year of observation.

The previously mentioned research conducted by Bower et al. also confirmed an amelioration in liver enzymes profile, including ALT (11.36 u/L, 95%CI 8.36–14.39), AST (3.91 u/L, 95%CI 2.23–5.59), ALP (10.55 u/L, 95%CI 4.40–16.70) and gamma-GT (18.39 u/L, 95%CI 12.62–24.16) [19]. A study conducted by Kirkpatrick et al. revealed a reduction in liver enzymes including ALT (66.21 vs. 28.58) and AST (46.28 vs. 24.69) during 12 months of observation [32]. Groth et al. also observed an amelioration in the liver enzymes profile in patients undergoing laparoscopic sleeve gastrectomy during 6 months of follow up (AST 22.0 (19.0–28.0) vs. 16.0 (13.0–22.0), *p* < 0.001, and ALT 27.5 (20.5–41.0) vs. 19.0 (15.0–27.0), *p* < 0.001) with no statistical differences regarding gender (*p* = 0.840) [33]. Similar results were observed in our study. We noted a statistically significant reduction in AST, ALT, GGT and LDH serum activity. A reduction of transaminase levels decreases the risk of progression to fibrosis and the end stage of liver disease. Additionally, Lee et al. proved that patients with elevated serum aminotransferase levels are at a higher risk not only of liver disease but also of all-cause mortality [34].

Nascimento et al. analyzed changes in NAFLD Fibrosis Score before and after bariatric surgery. The NAFLD Fibrosis Score changed from −0.6845 before the surgery to −1.6898 12 months after the procedure (*p* < 0.0002), indicating an absence of advanced liver fibrosis in any patient 12 months after the surgery [35]. An intermediate degree of fibrosis was identified in 12 patients (46.2%) one year after the bariatric procedure. The research conducted by Yang et al. also revealed statistically significant changes in the NAFLD score (−1.636 vs. −2.123, *p* < 0.001) over a two-year observation period [36]. Sandvik et al. observed a significant overall shift towards lower risk categories of advanced hepatic fibrosis based on NAFLD Fibrosis Score in 11.6 years of observation (NAFLD Fibrosis Score −1.32 (IQR −2.33; −0.39) vs. −1.71 (IQR −2.49; −0.95, *p* < 0.001) 11.6 years after surgery). In the above-mentioned study, a weak negative correlation between the decrease in NAFLD Fibrosis Score and weight loss parameters (%EWL (r = −0.251, *p* < 0.0001) and %TWL (r = −0.280, *p* < 0.0001)) was observed [37]. In our study, a statistically significant decrease in NAFLD Fibrosis Score was also seen. Additionally, we found a moderate negative correlation between NAFLD Fibrosis Score and weight loss parameters, including the percentage of total and excess weight loss and the percentage of excess BMI loss. Salman et al. analyzed patients with NASH-related liver cirrhosis of Child class A scheduled for laparoscopic sleeve gastrectomy due to morbid obesity. In their observation, the fibrosis score regressed to F2 in 19 patients (26.8%) and F3 in 29 (40.8%) during 30 months of follow up. Additionally, patients with improved Fibrosis Score had significantly higher weight loss (*p* <0.001). Thirty months after surgical treatment, 53.8% of cases with borderline NASH and 36.8% of those with probable NASH showed complete resolution. This study proved that bariatric surgery may be an option in patients with NASH-related hepatic fibrosis and morbid obesity [38]. In a study conducted by Murakami et al., the NAFLD activity score was reduced in 10 of the 11 patients (90.9%), and there was a significant difference between before and 1 year after laparoscopic sleeve gastrectomy (*p* < 0.05). Non-alcoholic steatohepatitis was no longer demonstrated in 81.8% patients in liver biopsy 1 year after the surgery; however, the fibrosis stage did not significantly ameliorate 1 year after laparoscopic sleeve gastrectomy [39].

The main limitation of our study is the fact that postoperative liver steatosis was evaluated with ultrasonography and not by hepatic biopsy to examine histological features of NAFLD. Some researchers may question ultrasonography as an imaging tool to predict the presence and severity of liver steatosis based on the fact that it is a performer-dependent and subjective imaging method. Generalizability of our results could be also impaired by the low number of participants, and therefore it is important to remember that some patients undergoing laparoscopic sleeve gastrectomy will not experience the amelioration of liver steatosis during observation. The surgical procedure may not always improve the grade of hepatic steatosis, or, in rare cases, it may even worsen the condition of the liver. Additionally, longer observation could be performed in order to achieve strong evidence that LSG improves the course of NAFLD.

## 5. Conclusions

In conclusion, our study confirms the thesis that laparoscopic sleeve gastrectomy is an effective method for the treatment of NAFLD in morbidly obese patients. Weight loss induced by LSG resolved NAFLD in more than 50% of patients according to ultrasound features of steatosis in one year of observation. Laparoscopic sleeve gastrectomy led to significant decrease in liver enzymes concentration and a reduction in NAFLD Fibrosis Score. Considering the increasing global prevalence of NAFLD, laparoscopic sleeve gastrectomy may be a crucial method of treatment in patients with morbid obesity and hepatic steatosis.

## Figures and Tables

**Table 1:** The assessment of liver steatosis in abdominal ultrasound during one year of observation.

	Follow Up	0	6 Months	12 Months
Liver Steatosis Status				
Steatosis	Grade 0	N/A	20 (37%)	34 (62%)
	Grade 1	6 (11%)	20 (37%)	19 (35%)
	Grade 2	33 (60%)	13 (24%)	2 (4%)
	Grade 3	16 (29%)	2 (4%)	0
Partial remission		N/A	27 (49%)	16 (29%)
Total remission		N/A	20 (37%)	34 (62%)

**Table 2:** Results of bariatric effects in study group.

Variables	0	6 Months	12 Months	*p*-Value
BMI (kg/m^2^)	45.6 (42.5–50.2)	33.5 (29.4–35.8)	31.0 (27.5–34.5)	**<0.0001**
%TWL	N/A	29.2 (25.2–32.4)	32.5 (28.2–36.9)	**0.0003**
%EWL	N/A	53.5 (46.3–62.4)	61.8 (52.4–72.3)	**0.0013**
%EBMIL	N/A	61.8 (53.6–74.4)	71.0 (61.3–86.9)	**0.0036**

Values are expressed as median (IQR). BMI, body mass index; %EBMIL, percentage of excess BMI loss; %EWL, percentage of excess weight loss; %TWL, percentage of total weight loss; N/A, not applicable.

**Table 3:** Results of selected laboratory parameters during one year of follow up.

Variables	0	6 Months	12 Months	*p*-Value
ALB (g/dL)	3.8 (3.7–3.9)	4.0 (3.9–4.2)	4.0 (3.9–4.1)	**<0.0001**
PLT (×10^9^/L)	234.0 (20.5–274.0)	218.0 (190.0–276.0)	233.0 (200.0–268.0)	0.5600
FPG (mg/dL)	110.0 (94.0–130.0)	94.0 (89.0–99.0)	89.0 (83.0–96.0)	**<0.0001**
Bilirubin (mg/dL)	0.6 (0.4–0.7)	0.8(0.5–0.9)	0.9 (0.6–1.1)	**0.0002**
GGT (IU/L)	28.5 (21.6–56.5)	18.0 (12.5–27.0)	18.0 (13.7–35.0)	**0.0003**
LDH (IU/L)	235.0 (186.0–271.0)	179.0 (154.0–203.0)	176.0 (152.0–184.0)	**<0.0001**
ALT (IU/L)	41.1 (21.0–53.9)	21.0 (14.7–26.0)	19.0 (16.0–24.0)	**<0.0001**
AST (IU/L)	25.5 (19.0–37.0)	18.1 (14.0–24.0)	20.0 (17.0–26.0)	**0.0002**
Total cholesterol (mg/dL)	178.0 (148.0–193.0)	178.0 (144.0–201.0)	180.0 (153.0–180.0)	0.8285
LDL (mg/dL)	114.4 (96.3–129.0)	106.4 (82.0–133.0)	113.5 (76.0–132.6)	0.6769
HDL (mg/dL)	45.8 (37.1–50.4)	47.5 (39.8–57.6)	54.0 (46.8–65.0)	**<0.0001**
TG (mg/dL)	156.1 (112.0–215.0)	109.0 (76.0–139.0)	86.0 (61.0–134.0)	**<0.0001**
NAFLD Fibrosis Score	0.2 (−0.8–1.0)	−1.1 (−2.3–−0.2)	−1.6 (−2.4–−0.4)	**<0.0001**

Values are expressed as median (IQR). ALB, serum albumin; PLT, platelet count; FPG, fasting plasma glucose; GGT, gamma-glutamyl transpeptidase; LDH, lactate dehydrogenase (LDH); ALT, alanine transaminase; AST, aspartate transaminase; LDL, low-density lipoprotein; TG, triglyceride; HDL, high-density lipoprotein.

## Data Availability

Not applicable.
